# Antiviral Activity of *Vitis vinifera* Leaf Extract against SARS-CoV-2 and HSV-1

**DOI:** 10.3390/v13071263

**Published:** 2021-06-29

**Authors:** Carla Zannella, Rosa Giugliano, Annalisa Chianese, Carmine Buonocore, Giovanni Andrea Vitale, Giuseppina Sanna, Federica Sarno, Aldo Manzin, Angela Nebbioso, Pasquale Termolino, Lucia Altucci, Massimiliano Galdiero, Donatella de Pascale, Gianluigi Franci

**Affiliations:** 1 Department of Experimental Medicine, University of Campania “Luigi Vanvitelli”, 80138 Naples, Italy; carla.zannella@unicampania.it (C.Z.); rosa.giugliano@unicampania.it (R.G.); annalisa.chianese@unicampania.it (A.C.); massimiliano.galdiero@unicampania.it (M.G.); 2 Institute of Biochemistry and Cell Biology (IBBC), National Research Council (CNR), 80131 Naples, Italy; carmine.buonocore@szn.it; 3 Department of Marine Biotechnology, Stazione Zoologica Anton Dohrn, Villa Comunale, 80125 Naples, Italy; giovanniandrea.vitale@szn.it; 4 Department of Biomedical Sciences, University of Cagliari, Cittadella Universitaria, 09042 Cagliari, Italy; g.sanna@unica.it (G.S.); aldomanzin@unica.it (A.M.); 5 Department of Precision Medicine, University of Campania “Luigi Vanvitelli”, Vico Luigi De Crecchio 7, 80138 Naples, Italy; federica.sarno@unicampania.it (F.S.); angela.nebbioso@unicampania.it (A.N.); lucia.altucci@unicampania.it (L.A.); 6 Institute of Biosciences and Bioresources (IBBR), National Research Council of Italy (CNR), 80055 Portici, Italy; pasquale.termolino@ibbr.cnr.it; 7 BIOGEM, 83031 Ariano Irpino, Italy; 8 Department of Medicine, Surgery and Dentistry “Scuola Medica Salernitana”, University of Salerno, 84081 Baronissi, Italy

**Keywords:** SARS-CoV-2, HSV-1, antiviral, antimicrobial, *Vitis vinifera*, leaf extract, natural extract, flavonoids, molecular networking, LC-MS

## Abstract

*Vitis vinifera* represents an important and renowned source of compounds with significant biological activity. Wines and winery bioproducts, such as grape pomace, skins, and seeds, are rich in bioactive compounds against a wide range of human pathogens, including bacteria, fungi, and viruses. However, little is known about the biological properties of vine leaves. The aim of this study was the evaluation of phenolic composition and antiviral activity of *Vitis vinifera* leaf extract against two human viruses: the Herpes simplex virus type 1 (HSV-1) and the pandemic and currently widespread severe acute respiratory syndrome coronavirus 2 (SARS-CoV-2). About 40 phenolic compounds were identified in the extract by HPLC-MS/MS analysis: most of them were quercetin derivatives, others included derivatives of luteolin, kaempferol, apigenin, isorhamnetin, myricetin, chrysoeriol, biochanin, isookanin, and scutellarein. Leaf extract was able to inhibit both HSV-1 and SARS-CoV-2 replication in the early stages of infection by directly blocking the proteins enriched on the viral surface, at a very low concentration of 10 μg/mL. These results are very promising and highlight how natural extracts could be used in the design of antiviral drugs and the development of future vaccines.

## 1. Introduction

Grapevine is one of the major fruit crops worldwide, in terms of economic value and cultivated area. A large amount is subjected to wine production; however, grape has become a model for molecular biology, genetics, and breeding, garnering significant research interest in different areas from the study of polyphenols [1,2,3] to the investigation of specific gene families, such as resistant genes [4].

Grapevine is a plant rich in bioactive compounds [5,6], known for its therapeutic effects. The grape extract was studied for a long time for its hepatoprotective [7], hypoglycemic [8], cardio-protective [9], antioxidant [10,11], antibacterial [11,12,13], and antiviral activity [11,14] which are due to the high levels of polyphenolic compounds found in grapes skin, seeds, and stem. The anti-cancer effect, that has long been studied over the years, is attributable to the consistent antioxidant activity of these molecules [15,16]. Resveratrol, for example, one of the most abundant molecules in the extracts, acts at various levels of carcinogenesis [17]. Grape seed extract inhibits the expression of the epidermal growth factor receptor (EGFR) in head and neck cutaneous squamous cell carcinoma [18], and of MEPK/ERK1-2 and MAPK/p38 in breast cancer, counteracting tumor invasiveness and progression. Some phenolic compounds, such as quercetin and catechins, given their similarity to estrogenic hormones, determine a pro- and anti-estrogenic receptor response in breast cancer [19]. Sharma et al. demonstrated the apoptotic activity of proanthocyanidins blocking COX-2 and the prostaglandin receptor (PGE2) [20]. In this context, two stilbenoids of wine, trans-astringin and trans-piceatannol, also show a cancer-chemiopreventive action by inhibiting cyclooxigenases and precancerous lesions [21]. Recently, Di Meo et al. evaluated the anti-cancer activity of grape seeds semi-polar extracts of two Italian grape varieties (Aglianico and Falanghina). They found that the extracts of both varieties are able to inhibit growth and migration of three different mesothelioma cell lines by activating apoptosis [22].

Antibacterial activity has widely been reported [23,24,25,26,27,28]. Andelkovic et al. [29] demonstrated that flavonols, phenolic acids, and flavan-3-ols were the main components of grape leaf extracts driving antioxidant and antibacterial activity, principally inhibiting Gram-positive bacteria. A similar effect has been observed by Rodriguez–Vaquero et al. [30], who analyzed the antimicrobial potential of three Argentinian wines: *Listeria monocytogenes* growth was highly reduced by caffeic acid, rutin, and quercetin. Instead, Papadopoulou et al. [31] reported that alcohol-free red and white wine extracts also had antimicrobial potential against *Escherichia coli* and *Candida albicans*, albeit it was less strong than that observed against *Staphylococcus aureus*.

Few studies have analyzed the antiviral activity owned by grapevine extract, mainly related to its potential of inducing disruption of the cell membrane and inhibiting the first phases of the viral life cycle, such as attachment and fusion to the host cell [32,33,34,35,36]. Matias et al. [37] compared the anti-adenoviral activity of Portuguese white-winemaking by-products and resveratrol, used as a standard phenolic compound, and very interestingly, the wine extract was able to inhibit adenovirus-5 replication after 24 h post-infection differently from resveratrol. Other studies reported the antiviral potential of grape seed extract (GSE) against Hepatitis C virus (HCV) [38]. Both the crude extract and phenolic compounds caused a significant reduction of HCV replication, as observed by the decrease of gene expression levels in Real-Time PCR. Furthermore, Su et al. [39] described the virucidal activity of GSE against Hepatitis A virus (HAV) and human enteric virus surrogates: the viral replication decreased considerably after treatment at 37 °C with 0.25, 0.50, and 1 mg/mL GSE. Also, the antiherpetic and anti-parainfluenza activities of *Vitis vinifera* (*V. vinifera*) leaves were investigated [38], indicating that fractions of different polarity also had different antiviral potential. The fractioned content derived from chloroform extract showed higher inhibition capability, both against Herpes simplex virus type 1 (HSV-1) and Parainfluenza virus (PIV). Very recently, we characterized the phenolic constituents, antioxidant, antibacterial, and, mainly, the antiherpetic activity of grape cane extracts derived from typical cultivars of Southern Italy [11], i.e., Greco, Aglianico, and Fiano. We prepared various extracts in a range of different pHs (1–13), demonstrating that the extraction at pH 13.00 was the optimal condition, since the phenolic yield was the highest. We described the presence of 75 compounds with different abundance profiles at various pHs and, interestingly, six of them were identified for the first time in grape canes. Furthermore, we evidenced that extracts at pH 7.00 and 13.00 were remarkably efficient in the inhibition of the early stages of HSV-1 infection. These data are very promising, suggesting that grapevine extracts could be used as cheap and eco-friendly innovative antiviral agents to reduce viral contamination in food and other material intended for human use. In this scenario, we demonstrated that another important source of bioactive compounds, *V. vinifera* leaf extract, had a strong antiviral potential against two important human pathogens, i.e., HSV-1 and the pandemic and currently widespread severe acute respiratory syndrome coronavirus 2 (SARS-CoV-2). The herpetic infection represents one of the most common infectious diseases in humans. The virus can be classified into two types: HSV-1 and type 2 (HSV-2). It has been estimated that 3.7 billion people under the age of 50 (66.6% of the world’s population) are infected by HSV-1 [40]; moreover, the World Health Organization (WHO) has observed that 491.5 million people aged 15–49 years (13.2%) have HSV-2 infection. HSV-1 is a common virus infecting humans that is generally transmitted by oral-to-oral contact, but also via the genital route [41,42]. It rarely can also be the causative agent of most severe and fatal diseases, such as keratitis, neonatal infections, and encephalitis. SARS-CoV-2 nowadays represents a serious problem for human health worldwide, both in economic terms and in terms of deaths caused, which to date amount to 3.5 million people [43]. It belongs to the *Coronaviridae* family, a well-known viral family containing other zoonotic viruses responsible for other pandemic outbreaks caused by SARS-associated coronavirus (SARS-CoV) and Middle East Respiratory Syndrome Coronavirus (MERS). The diseases are very similar and consist of severe acute respiratory infections in humans, characterized by fever, cough, shortness of breath, myalgia, and diarrhea [44]. Despite the advent of vaccines, deaths continue to increase. For these reasons, the identification of new promising drug candidates, especially of natural origin, that are able to interfere with SARS-CoV-2 and HSV-1 is essential.

## 2. Materials and Methods

### 2.1. Plant Material

Green healthy leaves were harvested in April 2019 in Portici (Naples, Italy) from a clone of *V. vinifera* (var. Paulsen 1103, kindly provided by Dr. Clizia Villano, from University of Naples “Federico II”). The plant has the following descriptive traits: apex is expanded, sublusty, green-bronzed with purplish edges. Apical leaves are showered with light velvet along the ribs of the lower page and are reddish green. The 4th to 6th leaflets are unfolded, whole, orbicular, have a U-shaped petiolar sinus with the base following the main veins, are bristly on the veins of the lower page, and are green with light bronzed reflections; ribs are reddish; petiole has short velvet. The plant stem is curved, ribbed, arachnoid, and purplish-red on one side. Herbaceous shoot is polygonal, with short velvet on the knots and has vinous-red internodes on one side; its knots are brown-purple. Tendrils are violet intermittent, bifid, and triffid; masculine flowers are present. Leaves are medium-sized, kidney-shaped, and whole, they have very open petiolar sinus with the base following the main ribs for a short distance; wavy flap is present; upper page is green, smooth, glabrous, and glossy; underside is light green and has bristly ribs; veins at the base of the upper page are pink; teeth are medium-sized, mucronate, and with convex margins, making it quite regular. Petiole is short, bristly, and vinous red on one side. Woody branch is of medium length and sturdiness, very branched, has a rounded cross section, an almost smooth surface, and a glabrous, grayish color with brown spots; internodes are medium distanced at 12–14 cm; buds are small. The plant blooms and leaves fall in the mid-late period. The plant has bushy habit, excellent vigor, rapid development, and good maturation of the wood; the plant is resistant to phylloxera and some fungal diseases. It is highly compatible with most grafting techniques and adapts to clayey-calcareous, slightly fresh, moderately chlorinated soils, and finally, it tolerates slightly brackish soils. A voucher specimen of this clone is kept and propagated in Portici. Growth conditions are normal open field conditions of the Campania (Italy) region. Fresh leaves were kept on ice and immediately freeze dried and pulverized with pestle and mortar.

### 2.2. Polyphenols Extraction

Polyphenols were extracted following the procedure developed by Docimo et al., 2016 [45] with some changes. A solution of 1.5 mL of 75% (*v/v*) methanol/0.05% (*v/v*) trifluoroacetic acid (TFA) was used for the extraction of 25 mg of pulverized leaf samples, by an incubation at room temperature for 1 h in continuous orbital shaker at medium speed. After sample centrifugation at 19,000× *g* for 10 min, and filtration by 0.2 mm polytetrafluoroethylene filters, the extract was concentrated in a Vacufuge Concentrator (Eppendorf, Hamburg, Germany) and lyophilized. The powder was solubilized at 100 mg/mL in DMSO: H_2_O (9:1). The weight ratio of the final lyophilized powder to the dried raw plant material was used to calculate the extraction yield.

### 2.3. LC-MS Analysis of Flavonoids

The extract was dissolved in MeOH at 5 mg/mL and an aliquot of 3 µL was analyzed through an Information Dependent acquisition (IDA) experiment conducted on a Q-TRAP™ 4500 (AB Sciex, Framingham, MA, USA) tandem mass spectrometer equipped with an ACQUITY UPLC BEH C18 column (130 Å, 1.7 µm, 2.1 mm × 50 mm) (Waters, Milford, MA, USA). Full MS spectra were acquired in Enhanced MS (EMS) mode in the *m/z* range 300–1000, then the 5 most intense ions were subjected to fragmentation in Enhanced Product Ion (EPI) mode. The EPI was conducted in the *m/z* range 50–1000 with a collision energy (CE) of −40 eV. Both EMS and EPI were conducted in negative mode, with a scan rate of 10,000 Da/s, with the source temperature at 200 °C, capillary voltage of 4.5 kV, and de-clustering potential at −150 V.

The gradient was run at a flow of 200 µL/min, using H_2_O and acetonitrile, as solvent A and solvent B, respectively, both were supplemented with 0.01% of formic acid. The adopted gradient was set as follows: from 5% to 30% B in 20 min, isocratic flow at 30% B for 1 min, from 30 to 100% B in 5 min, and isocratic flow at 100% B for 3 min.

### 2.4. Molecular Networking and Spectral Library Search

A molecular network was created with the Feature-Based Molecular Networking (FBMN) workflow [46] on GNPS [47,48]. Firstly, the raw MS data were converted from *.wiff to *.mzXML extension through the tool MSconvert tool by ProteoWizard, and secondly processed with MZmine in order to clean the signals, delete the noise, and calculate the peak’s area of each compound [43]. The networking description and parameters were automatically generated from GNPS; here, we present a brief extract. The output files were exported to GNPS for FBMN analysis, and the parameters were settled in accordance with the data as follows: the tolerance of the precursor ion mass was set to 2 Da and the tolerance of the MS^2^ fragment ion was set to 0.5 Da. The molecular network was created by setting the edges to have more than 3 matching peaks and a cosine score higher than 0.7. The maximum size of a molecular family was set to 100. Analogues were searched for against MS/MS spectra within a range difference of 300 in the precursor ion value. Finally, the molecular network was visualized using Cytoscape. The full description and the GNPS job can be publicly accessed through the link: https://gnps.ucsd.edu/ProteoSAFe/status.jsp?task=5b3274bd0bbd442794c9bbb1721b92f7 (accessed on 28 June 2021).

The relative abundance of the described compound was also calculated. Briefly, the abundance of each polyphenol in Table 1 was calculated as the percentage of its area divided by the sum of all the polyphenols areas. The relative abundance of DGMGs was calculated in the same way but referring to their areas.

(1) $$Relative\;abundance=\frac{Polyphenol\;area}{Total\;polyphenols\;areas}\times 100$$

### 2.5. Cell Lines and Virus

Vero cell line (ATCC CCL-81), HCT-116 (ATCC CCL-247), A549 (ATCC CCL-185), MCF7 (ATCC HTB-22), H9c2 (ATCC CRL-1446), HepG2 (ATCC HB-8065), and Hacat (ATCC PCS-200-011) cells were purchased from American Type Culture Collection (ATCC, Manassas, Virginia, United States). Vero cells were grown in Eagle’s Minimal Essential Medium (EMEM) supplemented with 10% Fetal Bovine Serum (FBS), 100 mg/mL of streptomycin, 2 mM L-glutamine, and 100 IU/mL of penicillin-streptomycin solution in a humidified atmosphere with 5% CO_2_ at 37 °C. HepG2 carcinoma cells were propagated in RPMI-1640 Medium containing 4.5 g/L glucose supplemented with 10% FBS, 100 IU/mL penicillin-streptomycin, and 2 mM L-glutamine. The other cell lines were propagated in Dulbecco’s Modified Eagle’s Medium (DMEM) with 10% FBS, 2 mM L-glutamine and 100 IU/mL antibiotics. All the materials used for cell culture practice were purchased from Euroclone, Milan, Italy. HSV-1 (strain SC16), carrying a lacZ gene driven by the CMV IE-1 promoter to express β-galactosidase, was propagated on Vero cells monolayers. SARS-CoV-2 (clinical isolate, kindly provided by Lazzaro Spallanzani Hospital, Rome, Italy) was propagated on Vero cells as already reported [49]. All experimental work involving SARS-CoV-2 virus was performed in a biosafety level 3 (BSL3) containment laboratory.

### 2.6. Cytotoxicity Test

Next, 4 × 10^4^ cells/well were plated in a 24-well plate and treated for 48 h, with *V. vinifera* extract at 65, 125, 250, and 500 μg/mL, and with Vorinostat (SAHA) for 24 h, used as a positive control. The activity of these molecules against the cell viability was determined by the Thiazolyl Blue Tetrazolium Bromide [3-(4,5-dimethylthiazol-2-yl)-2,5-diphenyltetrazolium bromide] (Sigma-Aldrich, St. Louis, MO, USA) MTT assay. After treatment with natural extract and SAHA, 0.5 mg/mL of MTT water solution was added to the cells for 3 h. The purple formazan crystals, after removal of the supernatant, were solubilized in iso-propanol (Carlo Erba Reagents, Cornaredo, Italy) and the absorbance was read at a wavelength of 570 nm with a TECAN M-200 reader (Tecan, Männedorf, Switzerland).

### 2.7. Antiviral Activity

Vero were plated into 12-well cell culture plates (2 × 10^5^ cells/mL for each well) in culture medium. The next day, leaf extract was dissolved in MEM without FBS at the following concentrations: 0.625, 1.25, 2.5, 5, 10, 50, 100, and 500 µg/mL. The antiviral effect was measured following different schemes of treatment, as previously described [50]. The percentage of infectivity inhibition was calculated by counting the number of plaques obtained in the presence of extract with respect to those in CTRL− (only virus without extract). All experiments were performed in triplicate. Greco extract was used as a positive control for HSV-1 and SARS-CoV-2 antiviral assays [11].

### 2.8. Gene Expression

Vero were plated into 12-well cell culture plates (2 × 10^5^ cells/mL cells for each well) in culture medium. The next day, the monolayer was treated with leaf extract and HSV-1 and SARS-CoV-2, as described above. After 24 h, the total RNA was isolated using TRIzol reagent and quantified by measuring the absorbance at 260/280 nm (NanoDrop 2000, Thermo Fisher Scientific, Waltham, MA, USA). A total of 1 µg of total RNA was reverse transcribed to cDNA by 5× All-In-One RT MasterMix (Applied Biological Materials, Richmond, Canada). A quantitative polymerase chain reaction was run in triplicate using a CFX Thermal Cycler (Bio-Rad, Hercules, CA, USA). Then, 2 µL of cDNA were amplified in 20 µL reactions using BrightGreen 2× qPCR MasterMix-No Dye (Applied Biological Materials) and 0.1 µM of primer. Relative target Ct (the threshold cycle) values of UL54, UL52 and UL27 (for HSV-1), and S protein (for SARS-CoV-2) were normalized to GAPDH, as the housekeeping gene. The mRNA levels of cells treated with leaf extract were expressed using the 2^−ΔΔCt^ method [51]. Primers were purchased by Eurofins (Vimodrone, Milan, Italy) and their sequences are reported in Table 1.

## 3. Results and Discussion

### 3.1. Flavonoid Composition of V. vinifera Leaves

Negative ionization mode HPLC-MS/MS is one of the powerful methods to characterize flavonoids because it provides information on both aglycones and glycosidic conjugates that can differentiate isomers [47,52]. HPLC-MS/MS IDA analysis was carried out to investigate *V. vinifera* leaves metabolic profile.

**Figure 1:**
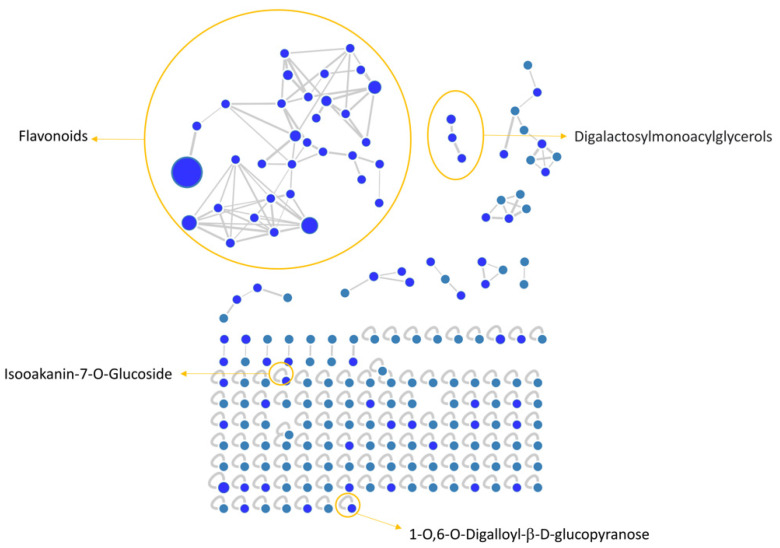
*V. vinifera* extract Feature Based Molecular Network (FBMN) obtained through GNPS analysis and Citoscape visualization, displaying the presence of flavonoids and digalactosylmonoacylglycerol clusters, together with two unpaired nodes matching with 1-O,6-O-Digalloyl-ß-D-glucopyranose and Isookanin-7-O-glucoside.

MZmine [53] and GNPS [54] software were used to convert and analyze the raw MS data, and finally Cytoscape built the complex network shown in Figure 1. Each node indicates a precursor ion, the node size is strongly related to its abundance in the sample (sum precursor intensity), and the edges size between two nodes is directly dependent on their MS/MS spectra similarity (cosine score) [55]. Analysis of GNPS results together with data manual curation evidenced the presence in the network of one big flavonoids cluster together with a smaller one belonging to digalactosylmonoacylglycerol (DGMG) lipids; other unpaired nodes have also been identified during GNPS analysis (Figure 1).

Assignment of each node has been manually curated and the putative identification is reported in Table 2. Results showed that compound **1** was not in a cluster and it was identified by the GNPS annotation tool as the tannin 1-O,6-O-Digalloyl-β-D-glucopyranose.

**Figure 2:**
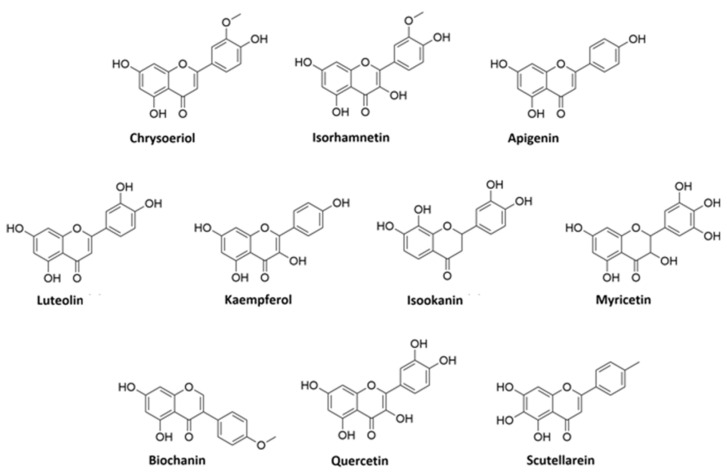
Structures of flavonoid aglycones identified in *V. vinifera*.

A detailed analysis of the MS and MS^2^ spectra, combined with the GNPS annotation tools, lead to the characterization of 35 flavonoids conjugates (see Table 2). Most of them were quercetin derivatives (8 compounds), others included derivatives of luteolin (5 compounds), kaempferol (4 compounds), apigenin (3 compounds), isorhamnetin (2 compounds), myricetin, chrysoeriol, biochanin, isookanin, and scutellarein (1 compound) (Figure 2). Four flavonoids were not identified (N.I.).

Moreover, we calculated the relative abundance of each compound present in Table 2 within the same chemical class. Results showed that compound **13** is the most representative among polyphenols, accounting for more than 44% of the total, followed by compounds **6** and **11**, with 16% and 13%, respectively. Compounds **8**, **14**, **15**, **26**, and **28** account for less than 10% each, while all the others are present in less than 1% or traces (Table 2). Among DGMGs, compound **37** is the most representative, with 97%, while compound **38** is present in traces and compound **39** represents 3% of the cluster.

### 3.2. Identification of Kaempferol and Luteolin Derivatives

The aglycone fragment at *m/z* 285 of compounds **2**, **20**, **23**, **25**, and **26** suggested that they originated from kaempferol or luteolin. Compounds **2**, **20**, and **26** produced a pseudo molecular ion at *m/z* 447 [M − H]^−^, and the MS^2^ profile showed typical fragments of hexose glycoside flavone, as they exhibited peaks at m/z 357 [M − H-90]^−^, 327 [M − H-120]^−^, and 297 [M − H-150]^−^. Thus, based on the MS^2^ fragmentation profile and RT, compound **2** could be assigned as kaempferol-8-C-glucoside, compound **20** as kaempferol-7-O-glucoside, and compound **26** as kaempferol-3-O-glucoside [52,56,57].

Compound **23**, *m/z* 593, is characterized by the neutral loss (NL) of (rhamnosyl-hexoside) moiety [M − H-308]^−^, and by comparison of its MS^2^ pattern with the literature, we assigned the identity as kaempferol-3-O-(6-O-rhamnosyl-galactoside) [58]. Compound **25** showed the NL of a glucuronide unit [M − H-176]^−^ and it was assigned as kaempferol-3-O-glucuronide.

Compounds **4**, **6**, **8**, **9**, and **18** are characterized by the presence of aglycone fragment at *m/z* 285 and they are all isomers with a parental ion at *m/z* 447 [M − H]^−^. These isomers were identified through the GNPS database and discriminated by RT as orientin, isoorientin, luteolin-4-O-glucoside, and ymaroside, respectively. Differently, compound **24** showed parental ion at *m/z* 483 and was generally assigned as luteolin-C-hexoside, by comparison with a precedent study [59].

### 3.3. Identification of Apigenin Derivatives

Compounds **10**, **11**, and **29** exhibited the same [M − H]^−^ ion at *m/z* 431. Compounds **10** and **11** showed a typical fragment of C-glycosides [M − H-120]^−^ at *m/z* 311. In addition, their MS^2^ pattern corresponds to the vitexin and isovitexin fragmentation profiles, respectively [58]. The isomer **29** was generally assigned as apigenin-C-glucoside.

### 3.4. Identification of Quercetin Derivatives

Compounds **13**, **14**, **17**, **19**, **21**, **22**, **31**, and **33** are all characterized by the presence of the quercetin aglycone at *m/z* 301. Compounds **13** and **17** showed parental ion at *m/z* 477 [M − H]^−^ with a NL of 176 Da [M − H-176]^−^ that is characteristic of the glucuronyl moiety. Compound **17** was identified by the GNPS assignment tool as quercetin-3-O-glucuronide, while its isomer, compound 13, was tentatively identified as quercetin-O-glucuronide [60]. Compounds **14**, **22**, and **31** showed the same pseudomolecular ion at *m/z* 463 [M − H]^−^, showing a NL of a glucoside moiety [M − H-162]^−^. GNPS assigned compound 14 as isoquercitrin, while compounds **22** and **31** were tentatively assigned as hyperoside and quercetin-7-O-glucoside, respectively, by the comparison of their RT and MS^2^ fragments with previous studies [58,61]. Compound **19**, *m/z* 505 [M − H]^−^, was identified by GNPS as Quercetin-3-O-glucose-6″-acetate, while its isomer compound **21** was generically assigned as quercetin-O-acetyl hexoside [60].

Compound **33**, *m/z* 639 [M − H]^−^, showed fragments at *m/z* 463 and 301 that correspond to the loss of glucuronyl and hexosyl moieties, respectively. Thus, on this evidence and coherently with a previous study [62], we assigned this compound as quercetin-O-glucuronide-O-hexoside.

### 3.5. Identification of Isorhamnetin Derivatives

Compounds **28** and **34** showed a fragment ion at *m/z* 315 that is characteristic of the isorhamnetin aglycone after the NL of a glucuronyl [M − H-176]^−^ and hexose [M − H-162]^−^ moiety, respectively. Coherently with the literature, we assigned compound **28** as isorhamnetin3(7)-O-glucuronopyranoside and compound **34** as isorhamnetin-O-glucoside [63].

### 3.6. Other Flavonoids Assignment

Compounds **5**, **15**, **16**, **32**, **35**, and **36** were identified by GNPS as myricetin-3-O-β-D-galactopyranoside, astragaloside, scoparin, 6-O-methylscutellarin, 4-(3,4-Dihydroxyphenyl)-5-beta-D-glucopyranosyloxy-7-methoxycoumarin, and isookanin-7-O-glucoside, respectively.

We could not assign any identification to compounds **3**, **7**, **12**, and **27**.

### 3.7. DGMG Assignment

Compound **37** was assigned as digalactosylmonoacylglycerol (DGMG) 18:3 by GNPS annotation tool. By comparison, compounds **38** and **39** were tentatively assigned as DGMG 20:5 and DGMG 16:2, respectively.

### 3.8. Antiviral Activity

**Figure 3:**
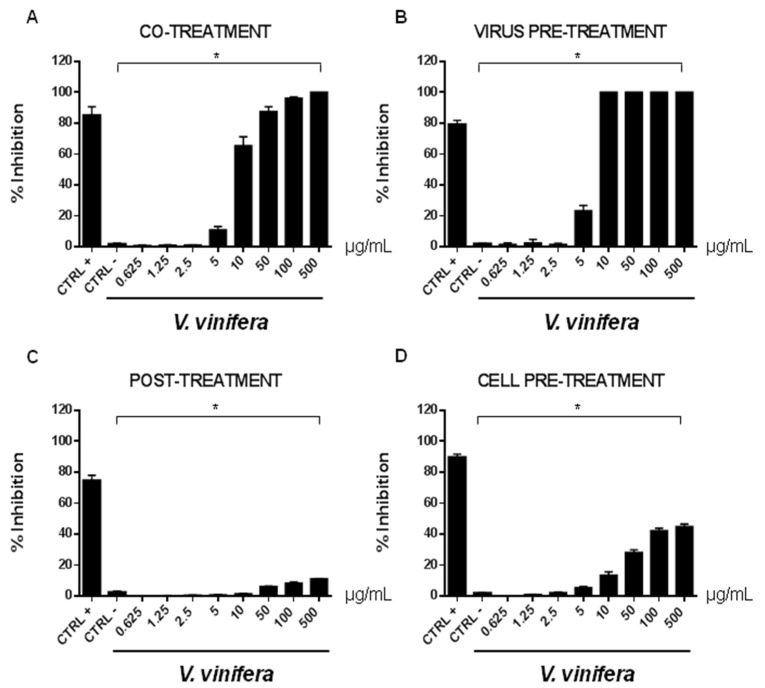
Antiviral effect (expressed as % of inhibition) of leaf extract (μg/mL) against HSV-1 in different plaque reduction assays: (**A**) co-treatment; (**B**) virus pre-treatment; (**C**) post-treatment; (**D**) cell pre-treatment. The results presented were obtained from three independent experiments. Data are mean ± SD. Statistical differences were analyzed via Student’s *t*-test, a value of *p* ≤ 0.05 was considered significant, with * *p* ≤ 0.05.

**Figure 4:**
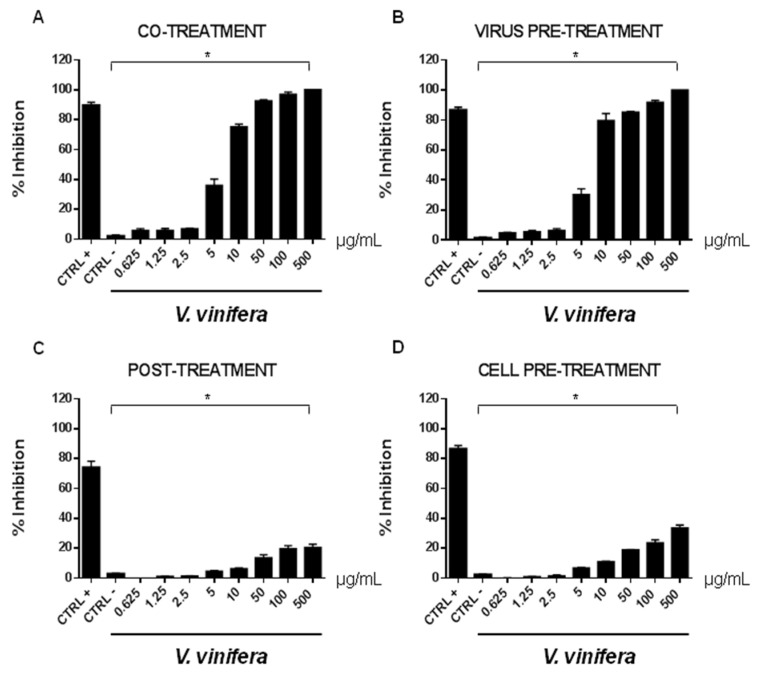
Antiviral effect (expressed as % of inhibition) of leaf extract (μg/mL) against SARS-CoV-2 in different plaque reduction assays: (**A**) co-treatment; (**B**) virus pre-treatment; (**C**) post-treatment; (**D**) cell pre-treatment. The results presented were obtained from three independent experiments. Data are mean ± SD. Statistical differences were analyzed via Student’s *t*-test, a value of *p* ≤ 0.05 was considered significant, with * *p* ≤ 0.05.

In order to evaluate if leaf polyphenol extract had an antiviral potential, and in which stage of the viral infection it could act, we performed experiments in four conditions: (a) simultaneous addition of extract and virus to the cells (“co-treatment”); (b) extract firstly incubated with the virus and then added to the cells (“virus pre-treatment”); (c) extract added to the infected cells (“post-treatment”); and (d) cells treated before with the extract and then infected with the virus (“cell pre-treatment”). The tested range of extract concentrations was from 0.625 to 500 µg/mL both for HSV-1 and SARS-CoV-2, which was used as a virus model for enveloped DNA and RNA viruses, respectively. The percentage of viral inhibition was calculated with respect to the untreated infected control (CTRL−). The reported antiviral activity was very similar for both viruses (Figure 3 and Figure 4), indicating an early effect manifesting directly on the viral envelope. The extract was most effective when incubated with HSV-1 and SARS-CoV-2 upon addition to target cells (co-treatment, Figure 3A and Figure 4A) or with the virus (virus pre-treatment, Figure 3B and Figure 4B) prior to infection on the cell monolayer. No activity was registered when the extract was added after HSV-1 infection (post-treatment, Figure 3C and Figure 4C), while a slight antiviral effect was observed when cells were firstly treated with the extract and then infected (cell pre-treatment, Figure 3D and Figure 4D). In detail, leaf extract was able to completely inhibit HSV-1 replication in virus pre-treatment assay until a concentration of 10 µg/mL was obtained but the virus resumed growing at 5 µg/mL, though with less efficiency (Figure 3B). Instead, the antiviral activity of *V. vinifera* extract was slightly lower against SARS-CoV-2, as it was set at 80% at 10 µg/mL. As HSV-1, no relevant inhibition was detected when the concentration of the extract was reduced to 5 µg/mL.

The leaf extract definitely showed a stronger antiviral potential than Greco extract, which was used as a positive control (CTRL+) against HSV-1 and SARS-CoV-2 [11,64,65,66].

**Figure 5:**
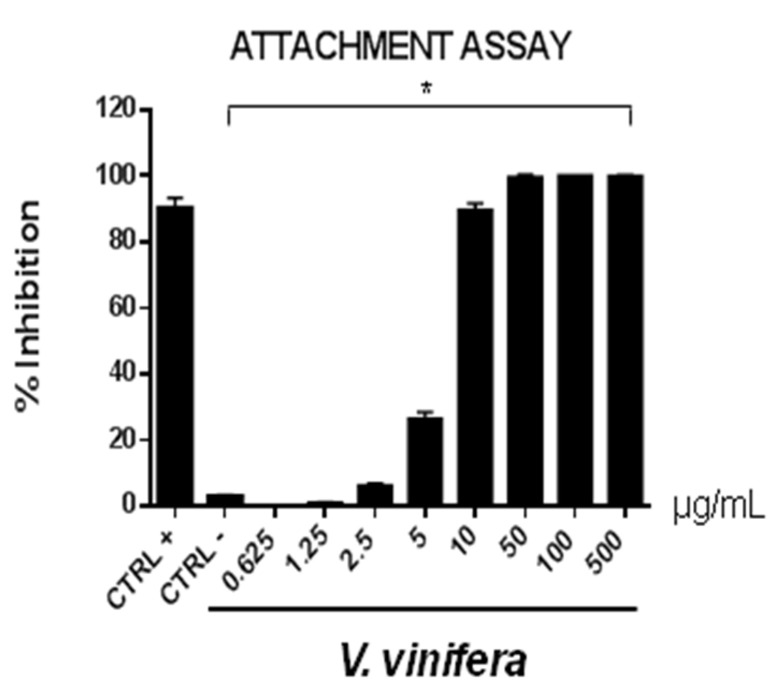
Early viral attachment assay of leaf extract (μg/mL) against HSV-1. The antiviral activity is expressed as % of inhibition. The results presented were obtained from three independent experiments. Data are mean ± SD. Statistical differences were analyzed via Student’s *t*-test, a value of *p* ≤ 0.05 was considered significant, with * *p* ≤ 0.05.

**Figure 6:**
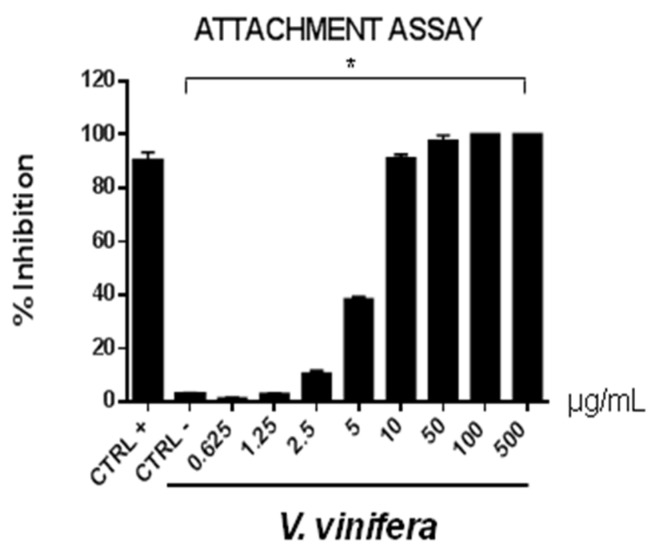
Early viral attachment assay of leaf extract (μg/mL) against SARS-CoV-2. The antiviral activity is expressed as % of inhibition. The results presented were obtained from three independent experiments. Data are mean ± SD. Statistical differences were analyzed via Student’s *t*-test, a value of *p* ≤ 0.05 was considered significant, with * *p* ≤ 0.05.

These data are very interesting and indicate that the leaf extract could act directly on viral particles, blocking the interaction with the cell membrane. To deepen the viral inactivation mechanism, another antiviral test was carried out, such as the attachment assay (Figure 5 and Figure 6), by shifting the temperature during the infection at 4 °C.

The extract’s inhibitory effects on virus-cell binding event revealed that it was able to block the attachment of both of the viruses into the host cell. It is most likely that leaf extract prevents the binding of viral envelope with the cell membrane and all the subsequent stages of infection by hindering some interacting site inside the viral glycoprotein deputed to the fusion, i.e., HSV-1 glycoprotein B and SARS-CoV-2 spike protein.

**Figure 7:**
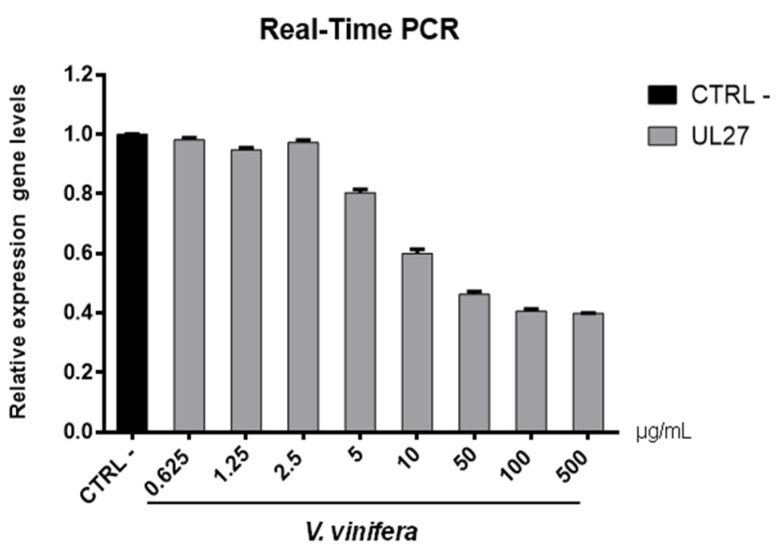
UL27 (glycoprotein B) gene expression analysis in Vero cells. *V.vinifera* leaf extract at 0.625, 1.25, 2.5, 5, 10, 50, 100, and 500 μg/mL was incubated with HSV-1 for 1 h and then, the mixture was added on Vero cells for another 1 h. After 2 days post-infection, RNA was extracted and Real-Time PCR was performed. Relative expression gene level was calculated with respect to CTRL−.

**Figure 8:**
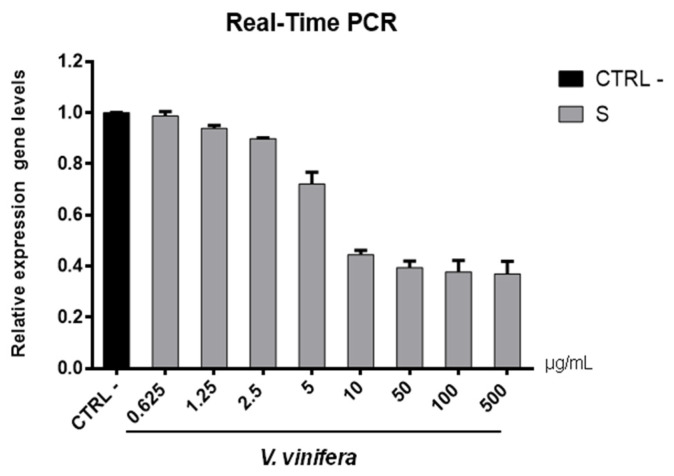
S gene expression analysis in Vero cells. *V.vinifera* leaf extract at 0.625, 1.25, 2.5, 5, 10, 50, 100, and 500 μg/mL was incubated with SARS-CoV-2 for 1 h and then the mixture was added on Vero cells for another 1 h. After 2 days post-infection, RNA was extracted, and Real-Time PCR was performed. Relative expression gene levels were calculated with respect to CTRL−.

To validate data obtained by plaque assays, we analyzed the *V. vinifera* effect on expression genes involved in the viral infection (Figure 7 and Figure 8).

Regarding the HSV-1 antiviral effect, UL27 gene, a late gene coding for the structural glycoprotein B (gB) was quantified via molecular assay (Figure 7). In brief, a virus pre-treatment assay was performed, as previously mentioned, and RNA was collected after 48 h post-infection. Results showed that the extract inhibited HSV-1 replication by blocking much of viral gene expression, which almost halved at a concentration of 10 µg/mL. The outcomes on the replication are in accordance with literature [67,68,69,70,71], demonstrating that the leaf extract had a virucidal action and could be used as a prophylactic treatment for herpetic infections. Anti-SARS-CoV-2 activity of *V. vinifera* leaf extract was also investigated through the spike protein (S) gene expression (Figure 8). S protein represents the anti-receptor inserted into the viral envelope essential to the attachment to the host cell via the human angiotensin-converting enzyme 2 (hACE2) receptor [72,73,74].

*V. vinifera* extract strongly reduced the S expression after SARS-CoV-2 infection until a concentration of 10 µg/mL was reached, and it resumed being expressed in a dose-dependent manner at lower concentrations. All together, these data indicated that *V. vinifera* leaf extract possesses a great antiviral and never previously investigated activity, which was reported against SARS-CoV-2. To date, the activity of flavonoids against SARS-CoV-2 has been analyzed with promising results in silico through bioinformatics approaches [46,48,75]; in addition, the activity of these molecules towards other coronaviruses, such as the Severe acute respiratory syndrome coronavirus (SARS-CoV) and Middle East Respiratory Syndrome Coronavirus (MERS), has been widely reported [76]. Altogether these results are noteworthy, although some phenolic compounds identified in the leaf extract are already reported as antiviral agents, such as quercetin which is currently under clinical trial [77]. This study focuses on the high anti-inflammatory ability of quercetin, effective in COVID-19 treatment, and its dosage depends on the type of treatment: 500 mg for prophylactic use and 1000 mg for curative treatment.

### 3.9. Cytotoxicity Evaluation

**Figure 9:**
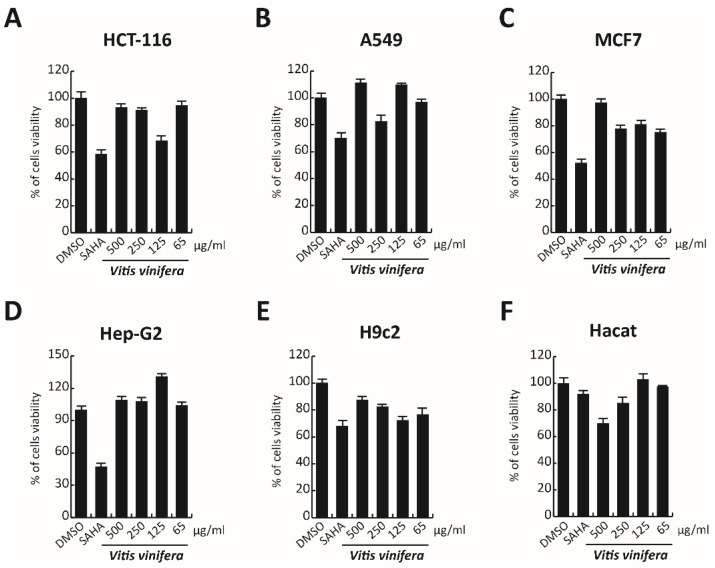
(**A**–**F**) Cell viability evaluation by MTT assay of HCT-116, A549, MCF7, cardiomyocytes, Hep-G2, and Hacat cells after treatment with *V. vinifera* for 48 h at 500, 250, 125, and 65 μg/mL. SAHA was used as a control at 5 μM for 24 h.

In order to complete the toxicological activity of leaf extract, *V. vinifera* was tested in colon, lung, breast, cardiomyocytes, hepatocyte cancer cells, and immortalized human keratinocyte cell line for 48 h at four different concentrations from 65 μg/mL to 500 μg/mL (Figure 9A–F). With respect to the known published anticancer effect of the grape, seeds, or peel of *V. vinifera* [78], the leaf extract did not induce any substantial variation in cell cancer viability. This different response, compared to that previously reported [79], could be explained by the different extraction methodologies or *V. vinifera* varieties used. These data suggest how this complex polyphenolic extract, in these conditions, was able to selectively act on a specific target, without altering other cellular mechanisms.

## 4. Conclusions

Plants represent an important source of natural products with high medical relevance [51]. Polyphenols, as flavonoids, are widespread compounds in the plant kingdom, and they are characterized by different biological activities, including antioxidant, antimicrobial, anticancer, anti-inflammatory, and antiviral properties. In this study, aqueous methanol solvent was employed to prepare raw extracts of *V. vinifera* leaves and its chemical profile was analyzed through HPLC-MS in negative ionization mode. This analysis led to the identification and characterization of 35 flavonoids, most of which were derivatives of quercetin. Others included derivatives of luteolin, kaempferol, apigenin, isorhamnetin, myricetin, chrysoeriol, biochanin, isookanin, and scutellarein. Furthermore, we tested the antiviral potential of the extract against HSV-1 and SARS-CoV-2 with very interesting results, showing the capability of flavonoids to inhibit SARS-CoV-2 for the first time. Considering the current pandemic emergency, our results represent a promising resource for pharmaceutical industrial applications.

## Figures and Tables

**Table 1:** Primers used in Real-Time PCR.

Gene	Sequence
HSV-1 UL27 forward	GCCTTCTTCGCCTTTCGC
HSV-1 UL27 reverse	CGCTCGTGCCCTTCTTCTT
SARS-CoV-2 S forward	AGGTTGATCACAGGCAGACT
SARS-CoV-2 S reverse	GCTGACTGAGGGAAGGAC
GAPDH forward	CCTTTCATTGAGCTCCAT
GAPDH reverse	CGTACATGGGAGCGTC

**Table 2:** Putative assignments of the nodes from the *V. vinifera* molecular network.

n.	Precursor Mass [M − H]^−^	Rt (min)	Putative Compound	Key Fragments	Relative Abundance
Polyphenols					
1	482.96	5.07	1-O,6-O-Digalloyl-β-D-glucopyranose	483; 301; 312; 271; 211	Traces
2	447.22	6.34	Kaempferol-8-C-glucoside	357; 327; 297; 285	Traces
3	789.63	6.63	N.I.	639; 613; 581; 477; 465; 463	Traces
4	447.16	7.60	Luteolin-8-C-glucoside (Orientin)	357; 339; 327; 299; 298; 297; 285	Traces
5	478.94	8.39	Myricetin-3-O-beta-D-galactopyranoside	479; 317; 316; 271; 270	0.1%
6	447.03	8.47	Luteolin-6-C-glucoside (Isoorientin)	357; 339; 327; 299; 298; 297; 285	16.0%
7	561.36	8.73	N.I.	449; 447; 357; 327; 301; 297	Traces
8	447.05	8.88	Luteolin-6-C-glucoside (Isoorientin)	357; 327; 299; 297; 285;	4.9%
9	447.00	9.16	Luteolin-4-O-glucoside	447; 327; 285; 284	Traces
10	431.03	9.45	Apigenin-8-C-glucoside (Vitexin)	377; 353; 341; 323; 311; 283; 282; 268	0.7%
11	431.06	9.85	Apigenin-6-C-glucoside (Isovitexin)	341; 323; 311; 293; 283; 281; 269	13.2%
12	499.07	10.02	N.I.	499; 323; 301; 300	0.1%
13	477.03	10.10	Quercetin-O-glucuronide	477; 301;	44.2%
14	463.07	10.30	Quercetin-3-O-glucoside (Isoquercitrin)	463; 301; 300; 271; 255	3.3%
15	447.06	10.62	Biochanin A-7-glucoside (Astragaloside)	447; 327; 285; 284	4.9%
16	461.06	10.81	Chrysoeriol 8-C-glucoside (Scoparin)	371; 341; 326; 313; 299; 298	Traces
17	477.09	10.83	Quercetin 3-O-glucuronide	477; 302; 301; 214	Traces
18	447.00	10.95	Luteolin-7-O-glucoside (Cynaroside)	447; 327; 285; 284	Traces
19	505.57	11.04	Quercetin-3-O-glucose-6″-acetate	505; 301; 300; 271; 255	Traces
20	447.09	11.27	Kaempferol-7-O-glucoside	447; 357; 327; 285; 284; 255; 256; 227	0.3%
21	505.37	11.29	Quercetin-O-Acetyl hexoside	302; 301; 300; 271; 255	Traces
22	462.98	11.31	Quercetin-3-O-galactoside (Hyperoside)	463; 301; 300; 271	Traces
23	593.15	11.54	Kaempferol-3-O-(6-O-rhamnosyl-galactoside)	285; 284; 257; 255	Traces
24	483.20	11.63	Luteolin-C-hexoside	483; 271	Traces
25	461.05	11.86	Kaempferol 3-O-glucuronide	285; 257; 228	0.6%
26	447.08	11.94	Kaempferol-3-O-glucoside	447; 327; 284; 285; 255; 256; 227	2.4%
27	517.06	12.35	N.I.	517; 355; 341	Traces
28	491.09	12.46	Isorhamnetin-3(7)-O-glucuronopyranoside	491; 315; 300	8.8%
29	431.01	12.95	Apigenin-7-O-glucoside	431; 283	Traces
31	463.11	13.45	Quercetin-7-O-glucoside	300; 301	Traces
32	475.06	14.34	6-O-Methylscutellarin	285; 284; 255; 256	0.3%
33	639.15	15.22	Quercetin-O-glucuronide-O-hexoside	463; 301	Traces
34	477.06	16.24	Isorhamnetin-O-glucoside	477; 315; 314	Traces
35	461.37	18.96	4-(3,4-Dihydroxyphenyl)-5-beta-D-glucopyranosyloxy-7-methoxycoumarin	299;284	Traces
36	449.26	19.90	Isookanin-7-O-glucoside	449; 431; 287; 269	Traces
	Digalactosylmonoacylglycerols				
37	675.35	24.81	DGMG 18:3	415; 397; 277; 235	97.1%
38	699.34	25.54	DGMG 20:5	653; 415; 397; 323; 255; 235	Traces
39	653.81	25.55	DMGM 16:2	653; 415; 397; 277; 255; 235	2.9%
